# Critically deviating vital signs among patients with non-specific diagnoses–A register-based historic cohort study

**DOI:** 10.1371/journal.pone.0293762

**Published:** 2023-11-01

**Authors:** Mia Carøe Sørensen, Morten Breinholt Søvsø, Erika Frischknecht Christensen, Tim Alex Lindskou

**Affiliations:** 1 Centre for Prehospital and Emergency Research, Aalborg University and Aalborg University Hospital, Aalborg, Denmark; 2 Department of Emergency and Trauma Care, Clinic of Internal and Emergency Medicine, Aalborg, Denmark; Kaohsuing Medical University Hospital, TAIWAN

## Abstract

**Background:**

One third of ambulance patients receive non-specific diagnoses in hospital. Mortality is 3–4%, however due to the high patient volume this group accounts for 20% of all deaths at day 30. Non-specific diagnoses do not provide much information on causes for death. Vital signs at first contact with ambulance personnel can act as a proxy for the patient’s condition. Thus, we aimed to describe the prevalence of abnormal vital signs, as determined by a modified NEWS2, in ambulance patients who received a non-specific hospital diagnosis. Secondly, we examined the association between vital signs, NEWS2 scores, type of non-specific diagnosis, and mortality among these patients.

**Methods:**

Register-based historic cohort study of ambulance patients aged 16+ in the North Denmark Region during 2012–2016, who received a non-specific diagnosis (ICD-10 chapters R or Z) at hospital. We used NEWS2 scores to determine if first vital signs were normal or deviating (including critical). Mortality was estimated with the Kaplan-Meier estimator. Association between vital signs and mortality was evaluated by logistic regression.

**Results:**

We included 41,539 patients, 20.9% (N = 8,691) had normal vital signs, 16.3% (N = 6,766) had incomplete vital sign registration, 62.8% (N = 26,082) had deviating vital signs, and of these 6.8% (N = 1,779) were critical. If vital signs were incompletely registered or deviating, mortality was higher compared to normal vital signs. Patients with critical vital signs displayed the highest crude 48-hour and 30-day mortality (7.0% (5.9–8.3) and 13.4% (11.9–15.1)). Adjusting for age, sex, and comorbidity did not change that pattern. Across all vital sign groups, despite severity, the most frequent diagnosis assigned was Z039 *observation for suspected disease or condition unspecified*.

**Conclusions:**

Most ambulance patients with non-specific diagnoses had normal or non-critical deviating vital signs and low mortality. Around 4% had critical vital signs and high mortality, not explained by age or comorbidity.

## Background

A substantial group of acute hospital patients do not receive a causal diagnosis, when assessed at the emergency department [1–4]. In studies from Scandinavia, around 30% receive a non-specific diagnosis, this also includes patients brought to hospital by emergency ambulance [5, 6]. These frequent non-specific diagnoses refer to the International Classification of Diseases 10^th^ Edition (ICD-10) chapters R (*symptoms*, *signs*, *and abnormal clinical and laboratory findings*, *not elsewhere classified*) and Z (*factors influencing health status and contact with health services*), thus patients with a symptomatology where no clear diagnosis such as an injury or infection could be assigned. Patients with non-specific diagnoses do not have a high mortality, but as a high number of patients regular receive these diagnoses, they account for 20% of the total number of deaths within 30 days among ambulance patients brought to a hospital in Denmark [5]. As a group, patients with non-specific diagnoses seem to encompass both low and high degree of disease severity, yet, perhaps due to unclear symptoms, specific diagnoses are not assigned at all, or later upon readmission [7]. So, non-specific diagnoses do not provide much information on the condition of the patient or the observed mortality. Patient vital signs can provide important information on exactly that.

Ambulance personnel will measure the patients’ vital signs as a part of the prehospital assessment [8], and through the Danish prehospital electronic medical record these vital signs will be sent to the emergency department staff within minutes. Using the first set of vital signs as part of an early warning score (EWS) can help determine the risk of poor patient outcome. EWS have the purpose of finding patients with clinical deterioration as early as possible allowing for faster intervention, and often consist of different components–most often patient vital signs–with different thresholds resulting in triage categories for how urgent action should be taken [9, 10]. A widely used and validated pre-hospital EWS is the National Early Waring Score 2 (NEWS2), including respiratory rate, oxygen saturation level, inhaled oxygen, body temperature, systolic blood pressure, pulse rate, and level of consciousness [11, 12]. Recent studies have shown that non-surviving patients have a significantly higher NEWS2-score compared to surviving patients [12–14] and the threshold for high clinical risk is defined as an aggregate NEWS2 of 7 or more [15].

Our objective was to report the prevalence of abnormal vital signs, as determined by a modified NEWS2, in patients who were brought to the hospital by emergency ambulance and later released with non-specific diagnoses. In addition, we examined the association between vital signs, modified NEWS2 scores, the type of non-specific diagnosis, and mortality among these patients.

## Methods

### Design

Register-based historic cohort study. We followed the STROBE guidelines for reporting of observational studies.

### Participants

Patients aged 16+ in the North Denmark Region calling the national emergency number, who were subsequently brought to a hospital by ambulance and received a non-specific primary diagnosis during 2012–2018 (2015 excluded). Patients with no valid civil registration number, or who received diagnoses associated with death at hospital arrival were excluded (see full list of diagnoses in S1 Table).

### Study setting

In Denmark, healthcare, including prehospital emergency medical services, is tax-supported with free and equal access for all citizens. The North Denmark Region constitutes approximate 10% of Denmark’s population (589,148 in 2018 [16]) covering primarily urban areas. The police handle all calls to the national emergency number (1-1-2), and forward calls of medical nature to the regional Emergency Medical Coordination Centre where health care professionals assess its urgency and severity using a criteria-based decision support tool [17] (Danish Index). All patients brought to a hospital receive a diagnosis according to the ICD-10 classifications. If a patient goes from one ward to another, they may receive additional or revise diagnoses.

### Outcome measure

We defined our primary outcome as mortality within 48 hours and 30 days stratified by severity of vital sign deviation.

### Variables and data sources

Data regarding patient vital signs (blood pressure, pulse, respiratory rate, SpO2 and Glasgow Coma Scale score) as well as ambulance dispatches, were obtained from the Prehospital Medical Record. We used the first registered measured vital signs, as a proxy for the patients’ medical condition when first seen by ambulance professionals.

Diagnoses according to ICD-10 were retrieved from the regional Patient Administrative System. The patients first received diagnosis in hospital was used, if a more organ specific diagnose were given during the patients’ hospitalization, they were not interpreted as having a non-specific diagnosis, and thereby excluded.

Finally, age, sex, and date of death were retrieved from the Danish Civil Registration System [18]. As the Danish Civil Registration System only contain a date of death, mortality within 48 hours was defined to ensure that patients who died within 24h of the emergency call, in case of incident crossing midnight, were included.

Data management and statistical analysis

### To assess patient vital signs, we applied a NEWS2 [15] inspired score, corresponding to the NEWS2 with body temperature omitted, as this is rarely measured in the ambulances (still referred to as NEWS2) [13, 19]

We defined the severity of vital signs according to this score as either *normal* (NEWS2 = 0) or *deviating* (NEWS2 not equal to 0) (see Table 1).

**Table 1:** Applied NEWS2 score. The modified NEWS2 score, with temperature omitted, used in the study.

NEWS2	3	2	1	0	1	2	3
Respiratory rate (bpm)	≤ 8		9–11	12–20		21–24	≥ 25
SpO_2_ Scale 1 (%)	≤ 91	92–93	94–95	≥ 96			
Air or oxygen		Oxygen		Air			
Systolic blood pressure (mmHg)	≤ 90	91–100	101–110	111–219			≥ 220
Pulse rate (bmp)	≤ 40		41–50	51–90	91–110	111–130	≥ 131
Consciousness (GCS)				15			< 15

Deviating vital signs were further subdivided into a *non-critical* (NEWS2 1–6) and *critical* (NEWS2 ≥7) group. Finally, we included a fifth group, compromised of patients with an *incomplete registration* of the NEWS2 score if any vital sign measurement were missing. Subcategory diagnoses were reported for each group. Mortality (48 hours and 30 days) was estimated for each of the groups and for most frequent subcategory diagnoses using the Kaplan Meier Estimator. We also evaluated the association between each vital sign group and mortality using logistic regression; crude, and adjusted for age, sex, and comorbidity. We used descriptive analysis to present patient characteristics and distribution with frequencies, medians and 95% confidence intervals (95% CI). Comorbidity was evaluated by Charlson Comorbidity Index (CCI) based on previous diagnoses (5 years) and grouped into 0, 1–2, 3+ [20]. Only observations with more than 5 patients were reported and all data was anonymized before statistical analysis in Stata/MP 17.0, so no individual patient could be identified [21].

### Ethics

The Danish Patient Safety Authority approved the study for the handover of medical records (ID 3-3013-1675/3). Furthermore, the study was registered in the North Denmark Region’s list of ongoing projects (ID 2020–067). According to Danish legislation, no patient consent or further approval (e.g., by an ethics committee), is required when approval for the handover of patient medical records has been given.

## Results

**Fig 1:**
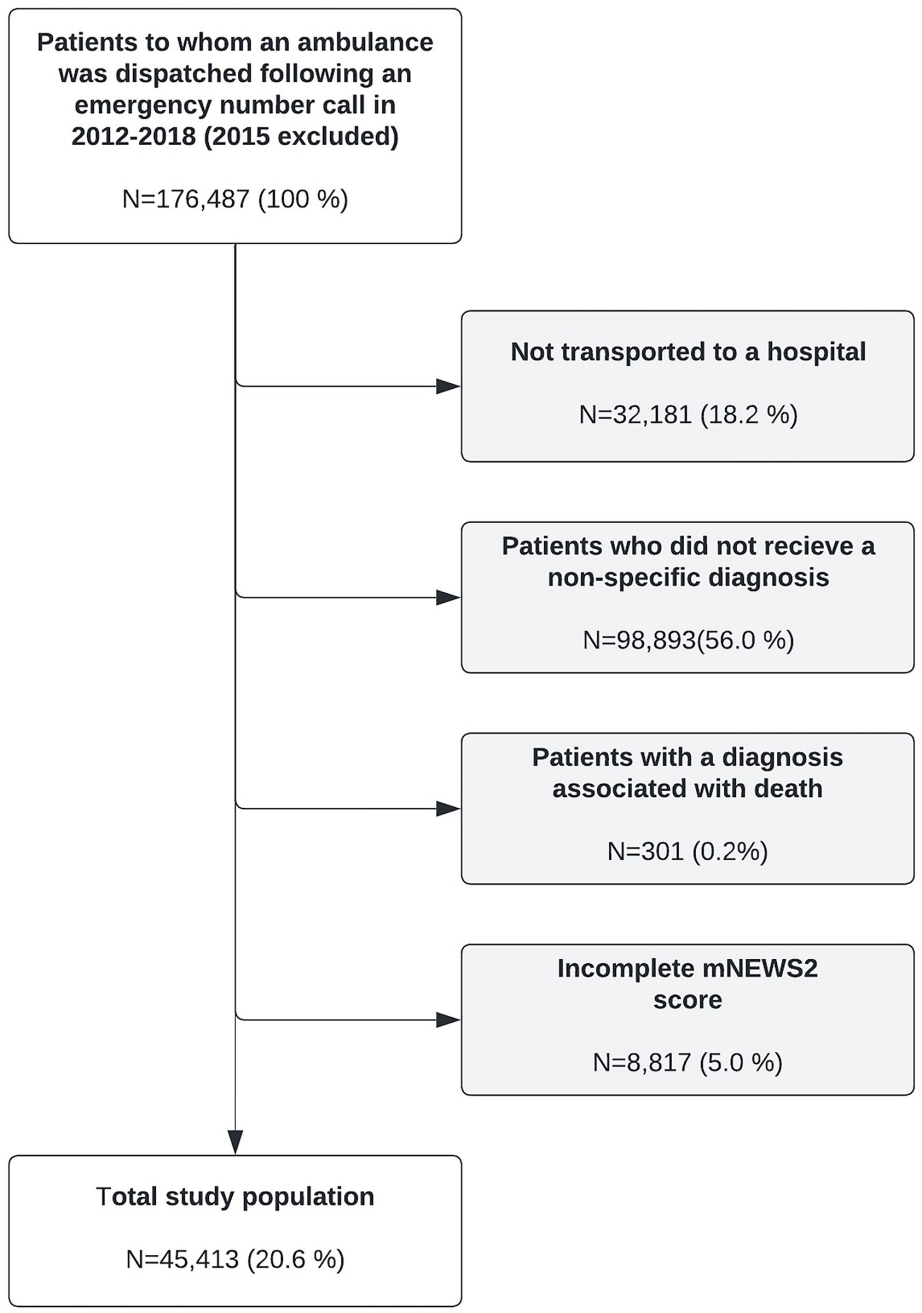
Flow chart. Patient contacts included in the study.

During 2012–2018 (excluding 2015) 176,487 patients had an ambulance dispatched in the North Denmark Region, following an emergency number call. The majority (144.316 (81.8%)) were brought to a hospital by ambulance, where 45,423 (25.7%) were assigned a non-specific diagnosis. Of these, 301 were excluded due to diagnosis indicating death at hospital arrival. Finally, 3,573 were under 16 years of age, resulting in the final 41,539 included patients (Fig 1).

### Vital signs, patient characteristics and diagnoses

Among all patients, 62.8% had deviating vital signs. The vast majority were non-critical (93.2%) while 6.8% patients had vital signs defined as critical in terms of severity (Table 2). Patients with critical vital signs were often male and older, compared to those with non-critical, incomplete registration, and normal vital signs. Patients with incomplete registration had the lowest median age of 53 years.

**Table 2:** Patient characteristics for each of the vital sign groups.

		Vital signs				
	Total	Normal	Incomplete registration	Deviating	Non-critical	Critical
**Total number (%)**	41,539 (100)	8,691 (20.9)	6,766 (16.3)	26,082 (62.8)	24,303 (93.2)	1,779 (6.8)
**Age, median (range)**	57 (16–103)	57 (16–101)	53 (16–98)	58 (16–103)	57 (16–103)	67 (16–102)
**Sex, number of females (% females)**	20,077 (48.3)	4,104 (47.2)	3,166 (46.8)	12,807 (49.1)	11,974 (49.3)	833 (46.8)
**Charlson Comorbidity Index score**						
0	26,985 (65.0)	6,124 (70.5)	4,508 (66.6)	16,353 (62.7)	15,542 (64.0)	811 (45.6)
1–2	11,027 (26.6)	2,063 (23.7)	1,757 (26.0)	7,207 (27.6)	6,551 (27.0)	656 (36.9)
>3	3,527 (8.5)	504 (5.8)	501 (7.4)	2,522 (9.7)	2,210 (9.0)	312 (17.5)

Most patients had no known comorbidity. Those with critical vital signs had higher degree of comorbidity; more than 1 in 6 patients had a CCI score of 3 or more (Table 2).

Regardless of whether patient vital signs were normal, deviating or incompletely registered, the diagnosis *Z039 Observation for suspected disease or condition*, *unspecified* was the most frequent followed by *R074 Chest pain*, *unspecified*. The most frequent remaining diagnoses were distributed as shown in S2 Table.

In the subgroup of patients with critical vital signs, certain diagnoses occurred more frequent; *R252A Convulsions* (7.4%), *unspecific*, *R060 Dyspnoea* (5.7%), *R559 Syncope or collapse* (5.2%) (S2 Table).

Pulse was the most frequent deviating vital sign with 42.7%, followed by oxygen saturation (26.6%) and respiratory rate (17.1%) (Table 3).

**Table 3:** Vital sign characteristics among patients with deviating vital signs (n = 26,082).

		NEWS2 determined value			
Vital signs	Median (IQR)	0	1	2	3
Respiratory rate	18 (16–20)	82.9	0.3	9.5	7.3
Oxygen saturation	97 (95–99)	73.4	12.8	5.1	8.4
Pulse	92 (77–105)	57.3	28.6	10.9	3.2
Systolic blood pressure	144 (127–163)	91.4	4.2	2.1	2.3
Level of consciousness	15 (15–15)	89.3	-	-	10.7

0 indicating no, 1 mild, 2 medium, and 3 severe deviating vital signs as determined in the NEWS2 score.

For patients with critical vital signs, oxygen saturation was the most frequent deviating vital sign (77.6%) followed by pulse (72.1%), and respiration rate (63.1%). In addition, for most of these patients, level of consciousness was affected (Table 4).

**Table 4:** Vital sign characteristics among patients with critically deviating vital signs (n = 26,082).

		NEWS2 determined value			
Vital signs	Median (IQR)	0	1	2	3
Respiratory rate	22 (18–26)	36.9	1.4	22.7	39.1
Oxygen saturation	91 (86–95)	22.4	12.9	13.1	51.6
Pulse	103 (80–123)	27.9	27.4	24.2	20.5
Systolic blood pressure	135 (109–158)	70.0	7.9	6.7	15.2
Level of consciousness	14 (12–15)	37.0	-	-	63.0

0 indicating no, 1 mild, 2 medium, and 3 severe deviating vital signs as determined in the NEWS2 score.

Respiratory rate was the most frequent missing vital sign with 10% missing among all included patients, whereas the remaining vital signs had 4–5% missing.

### Mortality

**Fig 2:**
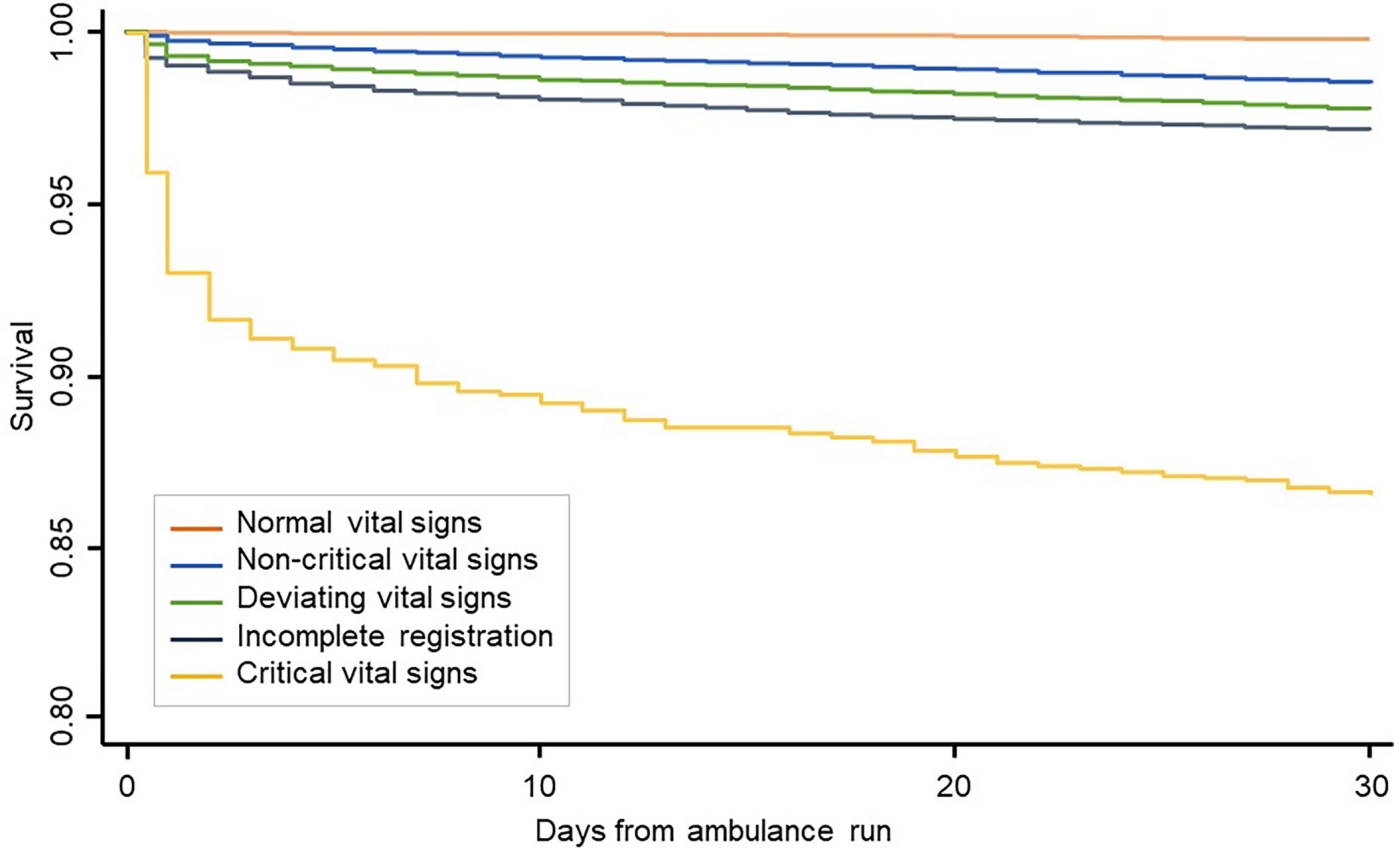
Mortality. Kaplan Meier plot showing the survival days after the ambulance run for patients categorized as normal-, non-critical-, deviating- and critical vital signs, as well as patients with incomplete registration of vital signs.

Compared to patients with normal vital signs, both 48-hour and 30-day mortality (crude) were higher among patients with incomplete registrations (P<0.001) and deviating vital signs (P<0.001). Patients with critical vital signs had a significantly higher mortality, compared to all other groups (P<0.001) (see Fig 2).

The differences in 48-hour mortality remained after adjusting for age, sex, and comorbidity with an odds ratio of 86.5 (95% CI: 12.0–623.8) for incomplete registration and 50.5 (95% CI: 7.1–360.5) for deviating vital signs (Table 5).

**Table 5:** Mortality within 48 hours and 30 days, with normal vital signs set as references. Reported as crude and adjusted for age, sex, and comorbidity.

	Odds ratio (95% CI)			
	Incomplete registration	Deviating vital signs	Non-critical vital signs	Critical vital signs
**48-hour mortality—crude**	85.6 (11.9–616.9)	59.4 (8.3–423.9)	19 (2.6–137.4)	651.1 (90.9–4662)
**48-hour mortality—adjusted**	86.5 (12–623.8)	50.5 (7.1–360.5)	17 (2.4–123.3)	409.9 (57.2–2939.1)
**30-day mortality—crude**	14.1 (8.7–22.8)	11.1 (6.9–17.7)	7 (4.3–11.2)	74.8 (46.2–121.1)
**30-day mortality—adjusted**	14.7 (9–24)	9.5 (5.9–15.2)	6.1 (3.8–9.9)	47.6 (29.2–77.5)

In the subgroups of the deviating vital signs, those with non-critical vital signs had a higher odds ratio of 17 (95% CI: 2.4–123.3) and those with critical vital signs had significantly higher with 409 (95% CI: 57.2–2939.1). This pattern was similar for 30-day mortality (Table 5).

The most frequent diagnosis, *Z039*, was assigned to more than half of all deceased patients, who died within 48 hours of the emergency call. No other single diagnosis accounted for more than 5 deaths.

At 30 days, *Z039* was assigned in nearly 45% of fatalities, followed by *R060 Dyspnoea*, accounting for approximately 5% (see S3 Table).

## Discussion

### Key findings

In this cohort study of 41,539 ambulance patients with non-specific diagnoses, there were three major findings. Firstly, most emergency ambulance patients assigned non-specific diagnoses at hospital release were not severely ill based on initial vital signs measured at ambulance encounter, and subsequent overall low mortality. Secondly, patients with deviating, incomplete, and especially critical vital signs, had increased odds of mortality which was not explained by age, sex, or comorbidity. Thirdly, *Z039 Observation for suspected disease or condition*, *unspecified*, was used most frequently across all patient groups, including patients with critical vital signs, where patients with Z039 exhibited substantial mortality.

### Comparison to other studies

Over a six-year period this study found 45,413 patients (27,6%) transported by ambulance to hospital and received a non-specific diagnosis, corresponding well with a previous Danish study [5] which found 31.6% in the same region. A British study [22] found an association between prehospital NEWS and 48-hours mortality in patients with critical vital signs (odds ratio 4.5) when compared to patients with non-critical vital signs. This supports our findings of higher mortality in patients with critical vital signs. In addition, Hoikka et al. [23] found that NEWS was associated with 1-day mortality in an unselected prehospital population. Hoikka et al. [23] also found a low mortality rate in patients with normal and non-critical vital signs which is in accordance with our results. Likewise, Ljunggren et al. [24] found an association between deviating vital signs measured in the emergency department and increased 1- and 30-day mortality and admission to intensive care unit. In the study by Ljunggren et al., the triage level (according to Rapid Emergency Triage and Treatment System—Adult) did not have the same associated odds for death as the individual vital signs did. Although the NEWS-score in the current study differs from the Rapid Emergency Triage and Treatment System—Adult, and the emergency department and prehospital setting does as well, it is possible that the same issues have occurred in the current study. However, the aggregated NEWS2 score of 7 or more managed to identify the group of patients in our population with the highest risk of poor outcome. In such patients, who are seemingly not severely ill, in the sense that they will receive a non-specific diagnosis upon release from hospital, the initial set of vital signs might still be useful to re-examine before sending the patient home. Patients with non-specific diagnoses might also be severely ill.

### Strengths and limitations of the study

The major strength of this study is the complete follow-up due to registry-linkage by the patient-unique civil registration number. Other strengths of this study are the large study population and the population-based design. The Danish citizens have free access to health care including prehospital emergency medical services minimizing selection bias based on financial resources. The North Denmark Region is comparable to the rest of Denmark, and a greater part of Scandinavia, providing external validity to the study. In interpretation of the findings of this study some limitations must be considered. One limitation is that only patients with known civil registration number were included which could lead to bias when these patients may be more or less ill compared to patients with unknown civil registration number. The modified NEWS2 score used in our study is not validated and was used as a pragmatic approach to assess the severity of the patients, with the most readily available routinely collected health data. The absence of temperature could have led to an underestimation of the aggregated NEWS2 score in our patient population leading to fewer patients in the critical vital sign group and more patients in the incomplete registration group. A Portuguese study [19] developing a short national early warning score, found that temperature did not contribute significantly to a predictive model for admission to an intensive care unit or death. As such, the omission of temperature in the current study, is expected to have had limited effect on the results regarding mortality.

In this study we did not include information regarding readmission, length of stay, and possible hospitalization. A previous study by Hansen et al. [7] shows that patients receiving the non-specific diagnosis Z03* at discharge had a lower 30-days mortality, but a higher readmission-rate compared to patients with a specific diagnosis at discharge. In addition, another recent Danish study [25] found that patients with non-specific diagnosis had a high number of readmissions, in general short length of stays, and the mortality were higher among patients with revisits.

## Conclusion

Ambulance patients with non-specific diagnoses encompass two patient groups: one very large group of patients with normal or non-critical deviating vital signs at ambulance encounter with corresponding low mortality, and a much smaller group of patients with critical vital signs at ambulance encounter with a high mortality, including older patients with more comorbidity. Most primary non-specific diagnoses assigned at hospital are similar regardless of vital sign severity, yet symptoms dyspnoea, convulsions, and syncope or collapse may require increased attention to patient outcome among patients with critical vital signs.

### Perspectives

In this study we have shown the importance of vital signs in a group of patients often looked to as not being critically ill. Indicating that if the first set of vital signs measured are deviating (especially critically deviating), patients with non-specific diagnosis will also be at increased risk of poor outcome. This may require increased focus at the emergency department assessment of the patient, both initially and later in the care pathway. Increased awareness on the initial state of the patient may be beneficial, as patients receiving non-specific diagnoses may be acutely ill or have existing disease as the reason for their acute condition.

## Supporting information

S1 Checklist STROBE statement—checklist of items that should be included in reports of observational studies. (DOCX)

S1 Table Diagnoses indicating death. List of diagnoses indicating death at hospital arrival and excluded from study population.
(DOCX)

S2 Table Diagnoses. The most frequent diagnoses, stratified by vital sign groups.
(DOCX)

S3 Table Diagnoses with most fatalities stratified by vital sign groups. Includes diagnoses where more than 5 deaths were present. 95% CI: 95% Confidence interval. Z039: Observation for suspected disease or condition, unspecified.
(DOCX)

## List of abbreviations

ICD-10: International Classification of Diseases 10^th^ Edition
EWS: Early Waring Score
NEWS: National Early Waring Score
NEWS2: National Early Waring Score 2
GCS: Glasgow Coma Scale
CCI: Charlson Comorbidity Index
