# Supplementary material for: Critically deviating vital signs among patients with non-specific diagnoses–A register-based historic cohort study
Source: PLoS One. 2023 Nov 1;18(11):e0293762. doi: 10.1371/journal.pone.0293762 (PMC10619789; doi:10.1371/journal.pone.0293762)
Supplement: S1 Table — List of diagnoses indicating death at hospital arrival and excluded from study population. (DOCX) [file pone.0293762.s002.docx]

| **Diagnoses associated with death at hospital arrival** |
| --- |
| 'R092 respiratory arrest', 'R96 sudden death',  'R96 sudden death', 'R99 certain circumstances regarding death’,  'R991 brain death according to the Danish Health Act §176’, 'R992 cardiac death according to the Danish Health Act §176' |

*Table S1. List of diagnoses indicating death at hospital arrival and excluded from study population.*
