# Supplementary material for: Critically deviating vital signs among patients with non-specific diagnoses–A register-based historic cohort study
Source: PLoS One. 2023 Nov 1;18(11):e0293762. doi: 10.1371/journal.pone.0293762 (PMC10619789; doi:10.1371/journal.pone.0293762)
Supplement: S2 Table — The most frequent diagnoses, stratified by vital sign groups. (DOCX) [file pone.0293762.s003.docx]

|  |  | **Vital signs** | | | | |
| --- | --- | --- | --- | --- | --- | --- |
| **Diagnosis (%)** | **Total** | **Normal** | **Incomplete registration** | **Deviating** | **Non-critical** | **Critical** |
| Z039 Observation for suspected disease or condition, unspecified | 10,599 (25.5) | 1,922 (22.1) | 1,735 (25.6) | 6,942 (26.6) | 6,296 (25.9) | 646 (36.3) |
| R074 Chest pain, unspecified | 4,300 (10.4) | 1,229 (14.1) | 488 (7.2) | 2,583 (9.9) | 2,528 (10.4) | 55 (3.1) |
| R559 Syncope or collapse | 1,903 (4.6) | 417 (4.8) | 246 (3.6) | 1,240 (4.8) | 1,147 (4.7) | 93 (5.2) |
| R429 Vertigo, unspecified | 1,110 (2.7) | 377 (4.3) | 147 (2.2) | 586 (2.3) | 572 (2.4) | 14 (0.8) |
| R100 Abdominal and pelvic pain | 1,322 (3.2) | 359 (4.1) | 203 (3.0) | 760 (2.9) | 746 (3.1) | 14 (0.8) |
| Z768 Persons encountering health services in other specified circumstances | 1,621 (3.9) | 283 (3.3) | 449 (6.6) | 889 (3.4) | 832 (3.4) | 57 (3.2) |
| Z038 Observation for other suspected diseases and conditions | 1,146 (2.8) | 195 (2.2) | 231 (3.4) | 720 (2.8) | 671 (2.8) | 49 (2.8) |
| Z035 Observation for other suspected cardiovascular diseases | 1070 (2.6) | 178 (2.1) | 125 (1.9) | 767 (2.9) | 731 (3.0) | 63 (2.0) |
| R252A Convulsions, unspecific | 935 (2.3) | 43 (0.5) | 166 (2.5) | 726 (2.8) | 595 (2.5) | 131 (7.4) |
| R060 Dyspnoea | 794 (1.9) | 71 (0.8) | 93 (1.4) | 630 (2.4) | 528 (2.2) | 102 (5.7) |
| Remaining | 16,739 (40.3) | 3,617 (41.6) | 2,883 (42.6) | 10,239 (39.3) | 9,657 (39.7) | 582 (32.7) |

*Table S2. The most frequent diagnoses, stratified by vital sign groups*
