# Supplementary material for: Critically deviating vital signs among patients with non-specific diagnoses–A register-based historic cohort study
Source: PLoS One. 2023 Nov 1;18(11):e0293762. doi: 10.1371/journal.pone.0293762 (PMC10619789; doi:10.1371/journal.pone.0293762)
Supplement: S3 Table — Includes diagnoses where more than 5 deaths were present. 95% CI: 95% Confidence interval. Z039: Observation for suspected disease or condition, unspecified. (DOCX) [file pone.0293762.s004.docx]

| **48-hour mortality**  **n deaths, mortality (95 % CI)** | **Normal** | | **Incomplete**  **registration** | | **Deviating** | | **Non-critical** | | **Critical** | |
| --- | --- | --- | --- | --- | --- | --- | --- | --- | --- | --- |
| *Z039* | 0 | | 36 | 3.4 (1.5-2.9) | 95 | 1.3 (1.1-1.6) | 29 | 0.4 (0.3-0.6) | 66 | 10.2 (8.1–12.8) |
| *Remaining* | Less than 5 | | 30 | 0.6 (0.4-0.8) | 82 | 0.4 (0.4-0.5) | 24 | 0.1 (0.0-0.1) | 58 | 5.1 (4.0-6-6) |
| **30-day mortality**  **(n deaths, mortality 95 % CI)** |  | |  | |  | |  | |  | |
| *Z039* | Less than 5 | | 89 | 2.1 (2.0-2.9) | 243 | 3.5 (3.0-4.0) | 131 | 2.1 (1.8-2.5) | 112 | 17.3 (14.6-20.5) |
| *R060 dyspnoea* | 0 | | 6 | 6.5 (3.0-13.8) | 40 | 6.4 (4.7-8.6) | 22 | 4.2 (2.8-6.3) | 18 | 17.7 (11.5-26.5) |
| *Remaining* | 16 | 0.2 (0.2-0.4) | 97 | 2.0 (1.6-2.4) | 303 | 1.6 (1.5-1.8) | 194 | 1.1 (1.0-1.3) | 109 | 10.6 (8.8-12.6) |

*Table S3: Diagnoses with most fatalities stratified by vital sign groups. Includes diagnoses where more than 5 deaths were present.
 95 % CI: 95 % Confidence interval. Z039: Observation for suspected disease or condition, unspecified.*
